# An EGR performance evaluation and decision-making approach based on grey theory and grey entropy analysis

**DOI:** 10.1371/journal.pone.0191626

**Published:** 2018-01-29

**Authors:** Xianghuan Zu, Chuanlei Yang, Hechun Wang, Yinyan Wang

**Affiliations:** Department of Power and Energy Engineering, Harbin Engineering University, Harbin, China; Southwest University, CHINA

## Abstract

Exhaust gas recirculation (EGR) is one of the main methods of reducing NO_X_ emissions and has been widely used in marine diesel engines. This paper proposes an optimized comprehensive assessment method based on multi-objective grey situation decision theory, grey relation theory and grey entropy analysis to evaluate the performance and optimize rate determination of EGR, which currently lack clear theoretical guidance. First, multi-objective grey situation decision theory is used to establish the initial decision-making model according to the main EGR parameters. The optimal compromise between diesel engine combustion and emission performance is transformed into a decision-making target weight problem. After establishing the initial model and considering the characteristics of EGR under different conditions, an optimized target weight algorithm based on grey relation theory and grey entropy analysis is applied to generate the comprehensive evaluation and decision-making model. Finally, the proposed method is successfully applied to a TBD234V12 turbocharged diesel engine, and the results clearly illustrate the feasibility of the proposed method for providing theoretical support and a reference for further EGR optimization.

## Introduction

Exhaust gas recirculation (EGR) is one of the effective ways to reduce NO_X_ pollutant emissions and has been widely used in marine diesel engines. The main process of EGR is to introduce a portion of the exhaust gas into the intake pipe, mix it with fresh air and enter the cylinder to re-enter the combustion process [1–3]. The key to EGR technology is to introduce a sufficient amount of exhaust gas into the intake pipe and provide the best EGR rate based on the different operating conditions of the engine [4–5]. Due to the differing effects of various EGR rates on diesel engine performance and emissions, the power, economy and emission performance of diesel engines must be considered when determining the optimal EGR rate. The basic principle of EGR is to reduce NO_X_ emissions as much as possible while having a minimal impact on other pollutant emissions. Most EGR has focused on ways of implementing EGR[6–7],experimental performance [8–9], EGR control [10–14] and no specific studies have evaluated EGR performance or the optimal EGR rate. Because of the complex operating characteristics of EGR under different working conditions, it is difficult to use classic assessment methods such a sanalytic hierarchy process, comprehensive index method, TOPSIS methods, to conduct unified assessments. In addition, most classic assessment methods are based on big data. However, there is no way of obtaining as much data as possible at times due to experiment condition limitations, which adds difficulty to EGR performance assessments. Therefore, developing reasonable assessment methods is an urgent need of EGR performance research.

In current EGR research involving turbocharged diesel engines, the problem of optimal EGR rate determination is only briefly introduced in some experimental studies. The most commonly used method is to develop different criteria through subjective judgment based on experimental data. Specifically, the main EGR parameters of diesel engines under different operating conditions must be obtained through a large number of tests. Then, different evaluation criteria are developed to determine the best EGR rate by different researchers through subjective judgment, Finally, an optimal EGR MAP can be obtained based on the working conditions. Although the criteria specified by different researchers vary depending on the focus of the study and subjective judgment, the basic principles are the same: to satisfy the NO_X_ reduction requirements for different conditions and simultaneously achieve good economic and emission performance. For example, Zhang [15] proposed that NO_X_ emissions meet the Tier III standard as a prerequisite for selecting the best EGR rate. Yang [16] proposed the criterion that the particle emission of13workingpointshould not exceed the emissions of the original machine in determining the best EGR rate. Han [17] proposed the strategy of optimizing NO_X_ emissions below 10% during peak smoke periods; as a result, the optimal EGR rate was obtained. Zhang [18] proposed that PM emission not exceed those of the original machine. Additionally, taking into account increased fuel consumption, decreased of NO_X_ emissions and other comprehensive factors, a high EGR rate should be selected at low-load conditions and a small EGR rate should be selected at high-load conditions. Through numerous calculations and analyses using BOOST model, Zheng [19] proposed a method of selecting the best EGR rate without overturning the torque, increasing the fuel consumption rate or increasing soot production. Then, the emission of 13-working-point and the optimal EGR rate were determined. Zhang [20] suggested that each load should correspond to the EGR rate that achieves the biggest decrease in effective fuel consumption, and this EGR rate can be defined as the optimum EGR rate. Other researchers such as Du [21] and Xu [22] employed similar methods.

All the above methods are intended to maximize diesel engines performance and effectively obtain a optimal EGR rate. However, these methods have some common shortcomings. First, each criterion is based on the researchers' subjective understanding of the performance improvement degree of the studied diesel engine. Although this professional expertise is invaluable, there is no specific mathematical support for this approach, and it is too subjective. In addition, these methods are not universal because of the different points of emphasis of the trials and different subjective judgments. Second, most of the methods rely on large quantities of test data. However, due to the limits of experimental conditions, sufficient experimental data are often unavailable. This limitation can inhibit the ability of decision makers to provide comprehensive judgments, and many existing method may not be applicable. Therefore, it is necessary to explore a new mathematical method from an objective point of view that considers the operating characteristics of EGR.

EGR performance evaluation can be viewed as a typical multi-objective decision-making problem. Therefore, the multi-objective grey situation decision-making method is used. As an important branch of grey theory, which is a classic artificial intelligence method, the multi-objective grey situation decision-making theory has unique advantages in decision-making problems for selecting the best scheme for a number of programs [23]. Because of its low computational complexity and high recognition effect, this method has been widely used in many fields, such as the choice of boost mode in turbocharged diesel engines [24], evaluations of the relationship between company attributes and financial performance [25], selection of waste disposal firms[26], evaluations of renewable energy sources [27] and other [28–30]. Currently, an increasing number of scholars have sought to optimize the decision-making model and improve the reliability of the results [31–36]. However, different optimization methods are limited to specific ranges and situations and not applicable to all decision-making issues. Performance evaluations and optimal rate determinations of EGR are strong complex and involves a trade-off between combustion and emission performance under different conditions. Traditional decision-making method sand the optimized methods proposed by different researchers cannot encompass the characteristics of EGR or satisfy the requirements of EGR optimization. A review of the relevant information indicates the lack of consensus on EGR performance evaluation and the optimal EGR rate of turbocharged diesel engines, and the related theoretical guidance is also lacking. Therefore, this subject must be further investigated. The objective of this study is to transform the characteristics and requirements of EGR into mathematical problems via a method that can be used for optimization in various e project applications.

In summary, this study proposes a multi-objective grey situation decision-making method based on the subjective and objective empowerment optimization to solve the problems associated with EGR performance evaluation and optimal decision making for turbocharged diesel engines. This method uses the advantages of the traditional grey decision, grey relational analysis, and grey entropy analysis while combining the characteristics and optimization requirements of diesel engine EGR performance, which can explore the intrinsic association between different EGR performance parameters and rank the different EGR schemes. The results demonstrate that this approach can be successfully applied to the EGR performance evaluation problem. The proposed approach has theoretical reference and guidance significance for optimizing the EGR performance of turbocharged diesel engines.

Compared to the relevant research, the major contribution of this paper is that for, EGR performance evaluation and optimal decision-making issues are evaluated for turbocharged diesel engine. An objective mathematical model and associated method are proposed to replace traditional subjective evaluation methods, and this new approach meets a major need in this field. In contrast to existing evaluation methods, the method proposed in this paper is applicable to both big data and cases with limited data. The optimization model is integrated with ideal EGR characteristics under different working conditions. This model yields higher modeling and simulation efficiency than traditional methods and satisfies the subjectivity and objectivity requirements; therefore, it is convenient for engineering applications. In addition, the method extends the engineering application scope of multi-objective grey decision-making, and also provides a new concept for EGR performance optimization of turbocharged diesel engines.

## Grey theory

Grey theory is mainly used to solve the uncertainty problem with limited data [23], and the multi-objective grey situation decision and grey relational analysis are among the two most important theoretical branches of grey theory. The mathematical model of the grey decision can be used to calculate the comprehensive effect measure of different schemes. The correlation coefficient and correlation degree between different sequences are calculated via grey relational analysis to achieve optimization [37–38].

### Multi-objective grey decision making

The main components of the traditional multi-objective grey decision model include the event set, strategy set, situation set, decision goal and decision weight.

First, construct the corresponding set of situations according to the event set and strategy set. Assume that *A* = {*a*_1_,*a*_2_⋯*a*_*n*_} is the event set, *B* = {*b*_1_,*b*_2_⋯*b*_*m*_} is the strategy set, *s* = {*s*_*ij*_ = (*a*_*i*_,*b*_*j*_)|*a*_*i*_ ∈ *A*,*b*_*j*_ ∈ *B*} is the situation set, and $u_{ij}^{\left(k\right)}(i=1,2\cdots ,n;j=1,2,\cdots m)$ is the effect sample value of the situation under the target.

Second, select targets to determine the effectiveness measure:

$$r_{ij}^{\left(k\right)}=\frac{u_{ij}^{\left(k\right)}}{\underset{i}{\max}\;\underset{j}{\max}\{u_{ij}^{\left(k\right)}\}}$$ (1)

The measure shown in Eq (1) is the upper effect measure and is mainly used to measure the degree to whichthe albino value deviated from the maximum whitening value.

$$r_{ij}^{\left(k\right)}=\frac{\underset{i}{\min}\;\underset{j}{\min}\{u_{ij}^{\left(k\right)}\}}{u_{ij}^{\left(k\right)}}$$ (2)

The measure shown in Eq (2) is the lower effect measure and is mainly used to measure the degree to which the albino value deviated from the lower limit.

$$r_{ij}^{\left(k\right)}=\frac{u_{i_{0}j_{0}}^{\left(k\right)}}{u_{i_{0}j_{0}}^{\left(k\right)}+\left|u_{ij}^{\left(k\right)}-u_{i_{0}j_{0}}^{\left(k\right)}\right|}$$ (3)

The measure shown in Eq (3) is the medium effect measure, where $u_{i_{0}j_{0}}^{\left(k\right)}$ is the moderate value of the specified effect under the target.

Each of these three measures is applicable to different situations. The upper effect measure is used to optimize for larger values, whereas the lower effect measure can be chosen to minimize loss. The medium effect measure is used if the desired outcome of the effect is to be near a specified value.

Third, solve the consistent effect measure matrix for the situation set based on the effect measure of each target.

$$R^{k}=(r_{ij}^{\left(k\right)})=\left[\begin{array}{cccc} r_{11}^{\left(k\right)} & r_{12}^{\left(k\right)} & \cdots & r_{1m}^{\left(k\right)}\\ r_{21}^{\left(k\right)} & r_{22}^{\left(k\right)} & \cdots & r_{2m}^{\left(k\right)}\\ \cdots & \cdots & \cdots & \cdots \\ r_{n1}^{\left(k\right)} & r_{n2}^{\left(k\right)} & \cdots & r_{nm}^{\left(k\right)} \end{array}\right]$$ (4)

where $r^{k}{}_{ij}=(r_{ij}^{\left(1\right)},\;r_{ij}^{\left(2\right)},\cdots ,\;r_{ij}^{\left(3\right)})$ is the consistent effect measure vector of situation *s*_*ij*_ under target k.

Fourth, establish the decision weight *η*_*k*_(*k* = 1,2,⋯*s*), where $\sum_{k=1}^{s}\eta_{k}=1$, and solve the integrated effect measure *r*_*ij*_ and integrated effect measure matrix of situation *s*_*ij*_.

$$r_{ij}=\sum_{k=1}^{s}\eta_{k}•r_{ij}^{\left(k\right)}$$ (5)

$$R=(r_{ij}^{})=\left[\begin{array}{cccc} r_{11}^{} & r_{12}^{} & \cdots & r_{1m}^{}\\ r_{21}^{} & r_{22}^{} & \cdots & r_{2m}^{}\\ \cdots & \cdots & \cdots & \cdots \\ r_{n1}^{} & r_{n2}^{} & \cdots & r_{nm}^{} \end{array}\right]$$ (6)

Finally, if $\underset{1\leq j\leq m}{\max\{r_{ij}\}}=r_{ij_{0}}$, then $b_{j_{0}}$ is the optimal strategy for event *a*_*i*_; if $\underset{1\leq j\leq m}{\max\{r_{ij}\}}=r_{ij_{0}}$, then $a_{i_{0}}$ is the optimal event for strategy *b*_*j*_; if $\underset{1\leq j\leq m}{\max\{r_{ij}\}}=r_{i_{0}j_{0}}$, then $s_{i_{0}j_{0}}$ is the optimal situation.

### Grey correlation analysis

Linear interpolation is used to transform the discrete behavior observations of the system factors into polylines of the segmented readings. Then, the model for the measure degree is constructed based on the geometric features of the polyline. The basic steps of the grey relational model are as follows:

Step1: specify the original sequence *X*_0_(*t*) = {*x*_0_(1),*x*_0_(2), ⋯,*x*_0_(*n*)} as the reference data sequence, also called the parent sequence, and *X*_*i*_(*t*) = {*x*_*i*_(1),*x*_*i*_(2), ⋯,*x*_*i*_(*n*)} as the sequence of data to be compared, also known as the subsequence.

Step2: make *ξ*_*i*_(T) the correlation coefficient for sequences *X*_0_(*t*) and *X*_*i*_(*t*) at time T:

$$\xi_{i}(T)=\frac{\underset{i}{\min}\;\underset{k}{\min}\left|x_{0}(T)-x_{i}(T)\right|+0.5\;\underset{i}{\max}\;\underset{k}{\max}\left|x_{0}(T)-x_{i}(T)\right|}{\left|x_{0}(T)-x_{i}(T)\right|+0.5\;\underset{i}{\max}\;\underset{k}{\max}\left|x_{0}(T)-x_{i}(T)\right|}$$ (7)

where 0.5 is the resolution factor and is typically between 0 and 1.

Step3: calculate the average of the correlation coefficients at each time of sequence *X*_*i*_(*t*), i.e., the degree of correlation of subsequence *X*_*i*_(*t*) to the parent sequence *X*_0_(*t*):

$$r_{i}=\frac{1}{N}\sum_{k=1}^{N}\xi_{i}(T)$$ (8)

## Optimization of the decision-making target weights

Although the traditional grey situation decision-making model has unique advantages in solving multi-attribute decision-making problems, it is limited in its ability to determine the decision-making target weight. In the traditional multi-objective grey decision model, the target weight is typically determined using the subjective weighting method. Although this method can serve the role of the expertise or the experience of experts or technical staff and has a certain degree of professionalism, it will impact the evaluation decision results because of its subjectivity and arbitrary nature. Therefore, this paper focuses on the optimization of the target weight. In view of the complexity of the EGR operational characteristics, neither the subjective weighting method nor the objective weighting method meet the requirements of EGR performance evaluation. Therefore, this paper adopts a subjective and objective comprehensive weighting method to optimize the decision-making target weight to consider the EGR performance optimization requirements as much as possible.

### Evaluation of the target selection

The first step of the proposed weighting method is to select the evaluation target. Due to the variable effects of different EGR rates on the combustion and emissions of diesel engines, the selection of the evaluation indicators should consider the combustion and emission performance of the diesel engine as much as possible. In this paper, the fuel consumption rate, in-cylinder explosion pressure, NO_X_, smoke and CO were selected as the evaluation targets. Because the main purpose of the EGR is to minimize the emission of NO_X_ pollutants, NO_X_ is defined as the main decision-making target, and the other four indicators are secondary decision-making targets.

The determination of the optimal EGR rate searches for the best compromise between the diesel engine combustion and emissions performances. The optimization of this compromise is reflected in the model as the primary consideration. Considering the important role of the target weights in the decision-making model, in the optimization, a compromise between diesel engine combustion and emission performance can be achieved by adjusting the target weight vector *η*_*k*_(*k* = 1,2,3,4,5), where k values of 1, 2, 3, 4, and 5refers to the fuel consumption rate, cylinder burst pressure, NO_X_, smoke and CO, respectively.

### Establishment of the initial subjective weight vector

a) Assign the main decision-making target weight by considering the main purpose of the EGR, which is to effectively reduce the NO_X_ emissions. Therefore, expert scoring is first used to customize the target weight for the NO_X_ emissions based on the variable conditions of the diesel engine. The following rules were established by repeating the trials and reviewing the acquired information:

I. When the diesel engine is under low-load conditions (defined as≤50% load), the NO_X_ emission concentration is low. A lower EGR rate should be chosen to ensure the stability and economy of the diesel engine, thus making the NO_X_ weight *η*_3_ = 0.4.

II. When the diesel engine is under high-load conditions (defined as ≥50% load), the NO_X_ emission concentration is high. A higher EGR rate should be chosen to ensure the necessary emissions, thus making the NO_X_ weight *η*_3_ = 0.5.

b) After determining the weight of NO_X_, the next step involves assigning the weights of the remaining indexes. Because each index represents a different aspects of diesel engine performance, relationships exist among indexes cannot be determined by direct observation or assessment. Therefore, grey relational analysis can be used as the basis for the distribution of weights.

The corresponding NO_X_ values (including the original machine value) at different EGR rates are used as the parent sequence, and the other four evaluation indicators corresponding to the value (including the original machine value) are used as subsequences. Then, the correlation coefficient $r^{'}{}_{i}(i=1,2,3,4)$ between the other four evaluation indexes and the NO_X_ index is solved by grey relational analysis. Finally, the correlation coefficient between the primary and secondary decision goals can be obtained as

$$r_{i}=\frac{r^{'}{}_{i}}{\sum_{i=1}^{4}r^{'}{}_{i}}$$ (9)

The correlation coefficient represents the degree of proximity of the secondary decision objective to the primary decision objective, which reflects their relative importance to the main decision-making targets.

c) Solve the initial subjective weight vector, defined as *η*_3_ and *r*_*i*_. The other four decision-making target weight values *η*_*k*_(*k* = 1,2,4,5) are determined using the formula *r*_*i*_(1-*η*_3_), and the initial subjective weight vector is ascertained.

### Establishment of the objective weight vector

The objective weight vector is based on the theory of uncertain decision-making combined with the characteristics of the EGR test data. Grey entropy analysis is introduced to explore the intrinsic relationship between each of the decision objectives, and the objective weight value is obtained.

In this paper, the event set *A* = {*a*_1_}, i.e., the event is the best EGR rate decision. The strategy set *B* = {*b*_1_,*b*_2_⋯*b*_*m*_} consists of *m* decision-making programs, and *b*_*m*_ represent different EGR rates.

According to the consistent effect measure matrix of each situation set for target k, $v_{1}^{(k)+}=m\underset{j}{a}xr_{1j}^{\left(k\right)}$ is defined as the positive ideal measure that represents the best test value for each index under current operating conditions, and $v_{1}^{(k)-}=m\underset{j}{i}nr_{1j}^{\left(k\right)}$ is defined as the negative ideal measure of target *k*, that represents the worst test value for each index under current operating conditions.

The deviations of the consistent effect measure $r_{ij}^{\left(k\right)}$ with its corresponding positive and negative ideal effect measures $v_{i}^{(k)+}$ and $v_{i}^{(k)-}$, respectively, are

$$e^{+}(r_{ij}^{\left(k\right)},v_{i}^{(k)+})=|r_{ij}^{\left(k\right)}-v_{i}^{(k)+}|e^{-}(r_{ij}^{\left(k\right)},v_{i}^{(k)-})=|r_{ij}^{\left(k\right)}-v_{i}^{(k)-}|$$ (10)

For event *a*_1_, the total deviation of the consistent effect under each strategy and target with its corresponding positive and negative ideal effect measures $v_{i}^{(k)+}$ and $v_{i}^{(k)-}$, respectively, can be expressed as a function of the target weight as

$$E^{+}(\eta_{k})=\sum_{j=1}^{m}\sum_{k=1}^{s}e^{+}(r_{1j}^{\left(k\right)},v_{1}^{(k)+})\eta_{k}E^{-}(\eta_{k})=\sum_{j=1}^{m}\sum_{k=1}^{s}e^{-}(r_{1j}^{\left(k\right)},v_{1}^{(k)-})\eta_{k}$$ (11)

Next, the weight *η*_*k*_ is determined such that the total deviation of the positive and consistent effect measures under each situation is minimized. The total deviation of the negative and consistent effect measures under each situation is maximized, which can be attributed to the following multi-objective optimization problem:

$$(\bar{P})\{\begin{array}{c} \min E^{+}(\eta_{k})=\sum_{j=1}^{m}\sum_{k=1}^{s}e^{+}(r_{ij}^{\left(k\right)},v_{i}^{(k)+})\eta_{k}\\ \max E^{-}(\eta_{k})=\sum_{j=1}^{m}\sum_{k=1}^{s}e^{-}(r_{ij}^{\left(k\right)},v_{i}^{(k)-})\eta_{k}\\ s.t.\sum_{k=1}^{s}\eta_{k}=1,\eta_{k}\geq 0,k=1,2,\cdots ,s \end{array}$$ (12)

Due to the uncertainty of the weight from the decision-making system with incomplete information, the uncertainty of the weight sequence should be minimized when determining the target weight. The target weight sequence is a grey connotation sequence *η*_*k*_(*k* = 1,2,⋯,*s*), and its grey entropy can be defined as

$$H_{⊗}(\eta )=-\sum_{i=1}^{s}\eta_{i}\ln\eta_{i}$$ (13)

According to the principle of maximum entropy, the determination of the target weight should minimize the uncertainty of the weight sequence. Therefore, the maximization constraint of the grey entropy is applied:

$$(\bar{P^{'}})\{\begin{array}{c} \max H_{⊗}(\eta )=-\sum_{i=1}^{s}\eta_{i}\ln\eta_{i}\\ s.t.\sum_{k=1}^{s}\eta_{k}=1,\eta_{k}\geq 0,k=1,2,\cdots ,s \end{array}$$ (14)

Then, $\left(\bar{P}\right)$ and $\left(\bar{P^{'}}\right)$ can be transformed into a single target optimization problem:

$$\begin{array}{l} \min\left\{\begin{array}{l} \mu \sum_{j=1}^{m}\sum_{k=1}^{s}e^{+}(r_{ij}^{\left(k\right)},v_{i}^{(k)+})\eta_{k}\\ -\mu \sum_{j=1}^{m}\sum_{k=1}^{s}e^{-}(r_{ij}^{\left(k\right)},v_{i}^{(k)-})\eta_{k}\\ +(1-2\mu )\sum_{i=1}^{s}\eta_{i}\ln\eta_{i} \end{array}\right\}\\ s.t.\sum_{k=1}^{s}\eta_{k}=1,\eta_{k}\geq 0,k=1,2,\cdots ,s \end{array}$$ (15)

where *μ* represents the balance factor between the three objectives and *μ* is typically 1/3.

Then, construct a Lagrangian function:

$$\begin{array}{l} L(\eta_{i},\lambda )=\mu \sum_{j=1}^{m}\sum_{k=1}^{s}e^{+}(r_{ij}^{\left(k\right)},v_{i}^{(k)+})\eta_{k}\\ -\mu \sum_{j=1}^{m}\sum_{k=1}^{s}e^{-}(r_{ij}^{\left(k\right)},v_{i}^{(k)-})\eta_{k}\\ +(1-2\mu )\cdot \sum_{i=1}^{s}\eta_{k}\ln\eta_{k}-\lambda (\sum_{k=1}^{s}\eta_{k}-1) \end{array}$$ (16)

Finally, *η*_*k*_ can be solved as

$$\eta_{k}=exp\left\{\begin{array}{l} {}[\lambda +\mu \sum_{j=1}^{m}e^{-}(r_{ij}^{\left(k\right)},v_{i}^{(k)-})\\ -\mu \sum_{j=1}^{m}e^{+}(r_{ij}^{\left(k\right)},v_{i}^{(k)+})] \end{array}/\left(1-2\mu \right)-1\right\}$$ (17)

### Establishment of the subjective and objective comprehensive weight vector

The objective weight vector *η*_*k*_(*k* = 1,2,3,4,5) and the initial subjective weight vector *η*_0*k*_(*k* = 1,2,3,4,5) are combined to obtain the subjective and objective comprehensive weight vector *η*_1*k*_(*k* = 1,2,3,4,5), which is typically obtained by the following formula:

$$\eta_{1k}=\frac{\eta_{k}\eta_{0k}}{\sum_{k=1}^{n}\eta_{k}\eta_{ok}}$$ (18)

## Establishment of the EGR evaluation and decision-making optimization model

For the EGR performance evaluation and decision making: event set *A* = {*a*_1_}, i.e., the event is the best EGR rate decision. Strategy set *B* = {*b*_1_,*b*_2_⋯*b*_*m*_} consists of *m* decision-making programs, and *b*_*m*_ represents different EGR rates. The decision-making evaluation targets represent the fuel consumption rate, in-cylinder explosion pressure, and the NO_X_, smoke and CO contents. Their corresponding weights are *η*_1_, *η*_2_, *η*_3_, *η*_4_ and *η*_5_.The possible conditions for each EGR rate are evaluated under the same experimental conditions, and $u_{ij}^{\left(k\right)}$ represents the measurement value corresponding to each decision objective under the various conditions for different EGR rates (that is, the experimental values when using different parameters). Lower values are better for the fuel consumption, cylinder burst pressure, NO_X_, CO and soot, and thus, a lower effect measure is chosen. The specific decision modeling steps are as follows:

Step1: develop the effect sample matrix $u_{ij}^{\left(k\right)}(i=1,2\cdots ,n;j=1,2,\cdots m)$, which is composed of the experimental data corresponding to different EGR rates under different working conditions. Then, solve the consistent effect measure matrix according to Eqs (1)–(3).

Because event *n* = 1 is used in this article, the effect sample matrix under different targets can be merged into a new matrix:

$$(u_{ij}^{})=\left[\begin{array}{cccc} u_{11}^{} & u_{12}^{} & \cdots & u_{1m}^{}\\ u_{21}^{} & u_{22}^{} & \cdots & u_{2m}^{}\\ \cdots & \cdots & \cdots & \cdots \\ u_{n1}^{} & u_{n2}^{} & \cdots & u_{nm}^{} \end{array}\right]$$ (19)

Where the abscissa *i* represents each target and the ordinate *j* represents different EGR rates.

Step2: solve the subjective and objective comprehensive weight vector *η*_1*k*_(*k* = 1,2,3,4,5) according to Eqs (9)–(18).

Step3: substitute *η*_1*k*_ into *(5)* to obtain the corresponding comprehensive effect measure matrix.

Step4: sort the advantages and disadvantages of the EGR schemes and optimize the EGR rate based on the principle of optimal decision.

## Test validation and result analysis

### Acquisition of test data

A model of a specific turbocharged diesel engine is used as the research object to verify the effectiveness of the optimization method. The main technical parameters of the diesel engine are provided in Table 1.

**Table 1:** Main technical parameters of the TBD234V12.

project	parameter
Power/kW	444(1800r/min)
Cylinderbore/mm×stroke/mm	128×140
Compression ratio	15: 1
Cylinder arrangement	V-shaped 12-cylinder 60° angle
Combustion chamber type	Direct injection w type

The test included the low, medium and high three-speed test, where the three speeds are 25%, 50%, and 75% load, respectively, under a total of 9 working conditions. A portion of the operating point test data is shown in Table 2. cgr, fc, co, no, soot and cbp represent the EGR rate, fuel consumption, CO, NO_X_, soot and cylinder burst pressure, respectively.

**Table 2:** A portion of the operating point test data.

	cgr	fc	co	no	soot	cbp
**OP1**	0	236.3	309	1093	0.045	7.6462
	2.2	241.6	316.57	1104.5	0.063	7.2545
	4.6	242.7	335.53	1002.6	0.088	7.2108
	7.5	243.9	366.7	943.5	0.084	7.1393
	9.8	246.9	427.84	890.65	0.12	7.0167
	11.5	248.7	503.62	783.6	0.27	6.9568
**OP2**	0	230.1	188	825	0.035	6.6859
	1.5	230.5	196.2	783.2	0.041	6.5428
	4.5	234.6	211.3	743.2	0.049	6.3595
	7.8	236.1	229.4	669.1	0.053	6.3052
	9.5	242.9	273.5	543.5	0.09	6.0485
	12.6	244.3	380.6	497.4	0.27	5.9274
**OP3**	0	257.5	180	810	0.035	8.2504
	2.2	256.7	196.2	832.45	0.051	7.7996
	4.5	259.9	211.3	786.3	0.055	7.6176
	7.1	261.3	229.4	701.6	0.06	7.5171
	9.3	266.8	273.5	643.5	0.078	7.4105
	11.8	268.1	380.6	589.7	0.19	7.1965
**OP4**	0	212.9	268	1438	0.108	9.5091
	1.7	214.7	286.53	1432	0.11	9.1568
	4.2	217.9	304.76	1351.6	0.13	9.0763
	7.4	218.2	329.89	1185	0.15	8.8016
	9.1	220.8	366.54	1069.4	0.21	8.7569
	11.8	224.3	426.71	994.2	0.33	8.5597
**OP5**	0	196.7	149	2012	0.125	9.2060
	2	196.9	156.4	1929.8	0.13	8.8482
	4.1	198.3	164.2	1853.8	0.152	8.6109
	7.9	203.1	172.2	1711.6	0.207	8.5634
	9.1	203.8	206	1534.6	0.28	8.4236
	11.2	208.9	312.3	1399.7	0.51	8.1596
**OP6**	0	200.4	160	2186	0.093	10.8
	1.6	199.8	156.4	2101	0.1	10.5505
	3.9	202.3	164.2	1894	0.13	10.4165
	7.5	205	172.2	1653	0.148	10.2256
	9.7	209.2	206	1521	0.165	10.0584
	11.1	212.2	312.3	1465	0.32	9.8568

### Analysis of the results

I.Low-load conditions

OP1 and OP2 represent the low speed at 50% load and medium speed at 25% load, respectively. Taking OP1 as an example, the EGR rates were 2.2%,4.6%,7.5%,9.8% and 11.5%. The effect sample matrix $u_{ij}^{\left(k\right)}$ is based on experimental data under different EGR rates:

$$(u_{ij}^{\left(5\right)})=\left[\begin{array}{l} 241.60\;242.70\;243.90\;246.90\;248.70\\ 7.2545\;7.2108\;7.1393\;7.0167\;6.9568\\ 1104.5\;1002.6\;943.50\;890.65\;783.60\\ 0.0630\;0.0880\;0.0840\;0.1200\;0.2700\\ 316.57\;335.53\;366.70\;427.84\;503.62 \end{array}\right]$$

Among these values, the abscissa represents the fuel consumption rate, CO, NO_X_, soot and in-cylinder burst pressure, respectively. The ordinate represents different EGR rates; for example, the first column represents an EGR rate of 2.2%.

Step 1: solve the consistent effect measure matrix:

$$(r_{ij}^{\left(5\right)}=\left[\begin{array}{l} 1.0000\;0.9955\;0.9906\;0.9785\;0.9715\\ 0.9590\;0.9648\;0.9744\;0.9915\;1.0000\\ 0.7095\;0.7816\;0.8305\;0.8798\;1.0000\\ 1.0000\;0.7159\;0.7500\;0.5250\;0.2333\\ 1.0000\;0.9435\;0.8633\;0.7399\;0.6286 \end{array}\right]$$

Step 2: solve the initial subjective weight vector; OP1 belongs to the low-load operating point, *η*_3_ = 0.4. Determine the grey association sequence:

Mother sequence:

X0=[10931104.51002.6943.50890.65783.60]

Subsequence:

X1=[236.3241.60242.70243.90246.90248.70]

X2=[7.64627.25457.21087.13937.01676.9568]

X3=[0.0450.06300.08800.08400.12000.2700]

X4=[309316.57335.53366.70427.84503.62]

The correlation coefficient between the other four evaluation indexes and the NO_X_ index are *r*_*i*_ = [0.2845 0.2915 0.2067 0.2173]. The initial subjective weight vector is *η*_*k*_ = [0.1707 0.1749 0.4000 0.1240 0.1304].

Step 3: solve the comprehensive weight vector. The objective weight vector is obtained through entropy analysis as *η*_0*k*_ = [0.1872 0.1816 0.1596 0.2445 0.2270]. The integrated weight vector is *η*_*k*_ = [0.1704 0.1694 0.3405 0.1617 0.1579].

Step 4: Solve the comprehensive effect measure matrix and sort the advantages and disadvantages according to the optimal principle, *R* = [0.9052 0.9027 0.9031 0.8735 0.8203]. The following performance ranking of the different EGR rates is obtained for OP1:2.2%>7.5%>4.6%>9.8%>11.5%. The optimal EGR rate is 2.2% for this condition, and when the EGR rate is less than approximately 8%, there is only a slight difference between the comprehensive evaluation values obtained with the different EGR rates. When the EGR rate increases, the comprehensive performance evaluation value decreases significantly. Therefore, a smaller EGR rate should be adopted.

The performance ranking of different EGR rates under OP2 and OP3can be obtained in a similar manner as follows:

OP2=[0.88430.89480.83450.78260.6974]

OP3=[0.93000.90310.88380.80950.7690]

4.5%>1.5%>7.8%>9.5%>12.6%

2.2%>4.5%>7.1%>9.3%>11.8%

The optimal EGR rates of theOP2 and OP3 conditions are 4.5% and 2.2%, respectively. When the EGR rate rises, the corresponding evaluation value decreases, and the decline is more significant when the EGR rate is greater than approximately 8%.

The results of OP1, OP2 and OP3 indicate that a lower EGR rate should be adopted when the diesel engine is under low-load conditions. The comprehensive evaluation value significantly decreased with increases in the EGR rate. The analysis of the reasons for this result indicated that when at low load, the NO_X_ pollutant emissions is low and a portion of the dynamic performance of diesel engines will be consumed if the EGR rate is excessively high. The EGR rate must be reduced to ensure the economy and power of diesel engines.

II.High-load conditions

OP4, OP5 and OP6 represent a 75% load at different speeds. Let *η*_3_ = 0.5; then, based on the simulation calculations, the comprehensive effect measure matrices of OP4, OP5 and OP6 are as follows:

OP4:[0.85060.84020.86660.86780.8606]

OP5:[0.83350.84940.87440.88860.8887]

OP6:[0.84860.85160.88740.88700.8239]

The performance ranking of the different EGR rates under OP3 and OP4 can be obtained as

OP4:9.1%>7.1%>11.8%>1.7%>4.2%

OP5:11.2%>9.1%>7.9%>4.1%>2%

OP6:9.7%>7.5%>3.9%>1.6%>11.1%

The results for OP4, OP5 and OP6 indicate that the comprehensive evaluation value of each EGR rate increases with increases in the EGR rate, and the change is significant when the EGR rate is greater than 8%. This result indicates that a lower EGR rate is less effective at improving the overall performance of diesel engines when the diesel engine is working at a high load. Furthermore, according to the results for OP6, when the EGR rate increases to 11.1%, the corresponding comprehensive evaluation value decreases. This trend occurs because OP6 is at high speed and high-load conditions to ensure adequate power performance; thus, a high EGR rate should not be used.

In summary, the above assessment and decision-making results demonstrate that when working under low-load conditions, because of the lower NO_X_ emission concentration, a smaller EGR rate should be used to balance the power and economy of diesel engines. When working under high-load conditions, the NO_X_ emission concentration is high and a higher EGR rate should be adopted to ensure the emissions performance. However, excessive EGR rates may have a negative impact on diesel engines when working under high-speed, high-load conditions. This finding is consistent with the characteristics of EGR performance for the current turbocharged diesel engine and demonstrates the effectiveness of the optimization method. However, compared to existing methods, the proposed method has a higher modeling and simulation efficiency and provides more reasonable results. Therefore, it can replace existing methods as a more convenient approach for engineering applications.

The EGR rate is limited to less than 15% in this study, and no further data can be obtained. However, the authors believe that this limit does not affect the main concept of this method because the core of this method is concerned with the EGR performance evaluation and decision-making problem with less data and more uncertainty, which is also the characteristic of this method that distinguishes it from the existing evaluation methods. Therefore, this method can provide effective theoretical support for the current EGR performance evaluation and decision-making of turbocharged diesel engines, which has certain guiding significance.

## Conclusion

Aiming at the problem of EGR performance evaluation and optimal decision-making for turbocharged diesel engines, the optimal compromise between the combustion and emission performance of the diesel engine is transformed into a weighting problem between the different evaluation targets. On this basis, a multi-objective grey situation decision-making method based on subjective and objective comprehensive weighting is proposed. This method can exert advantages in terms of subjective weighting and objective weighting and can integrate the EGR operating characteristics of the turbocharged diesel engine into the optimization model, which makes the final decision result more reasonable.

The results demonstrate that when the turbocharged diesel engine is under a low load, the difference between the comprehensive evaluation values of different EGR rates is not significant when the EGR rate is less than 8%, and the comprehensive evaluation value significantly decreases with increases in the EGR rate when the EGR rate is greater than 8%.Thusa lower EGR rate should be used. When the diesel engine is under a high load and the EGR rate increases to approximately8%, the corresponding comprehensive evaluation value significantly increases. Therefore, a higher EGR rate should be used. However, this increase is limited because excessive EGR rates may have a negative impact on diesel engines.

The results demonstrate that the decision results obtained using this method are consistent with the performance characteristics of the EGR as well as the current best EGR rate determination principles. Therefore, the proposed method can be successfully applied to determine the optimal EGR rate of turbocharged diesel engines under different conditions.

## Supporting information

S1 Test Data All relevant data can be found from the database of Chinese dissertations and the degree thesis title is"the calculation and experimental research with EGR system for V type diesel engine study,” which is shown in "TEST DATA.xlsx". (XLSX)
